# A case of COVID-19 vaccination during radiotherapy for breast cancer

**DOI:** 10.1186/s43046-022-00134-3

**Published:** 2022-08-15

**Authors:** Noriyoshi Takahashi, Kazuya Takeda, Yu Suzuki, Keita Kishida, Satoshi Teramura, Keiichi Jingu

**Affiliations:** 1 Department of Radiation Oncology, Tohoku University Graduate School of Medicine, Sendai, Japan; 2 Department of Radiation Oncology, South Miyagi Medical Center, Ōgawara, Miyagi Japan

## Abstract

**Background:**

The coronavirus disease 19 (COVID-19) vaccination has been progressing. The safety of vaccination during radiotherapy is not clear.

**Case presentation:**

We experienced a patient who received a COVID-19 vaccine during radiotherapy. A 60-year-old woman with breast cancer underwent postoperative radiotherapy. She received two vaccine doses and she suffered from severe vertigo. Her radiotherapy was suspended for several days and the radiotherapy schedule needed to be changed.

**Conclusions:**

The association between vertigo and vaccination during radiotherapy is not clear. However, if the general condition of patients worsens, suspension of treatment might be necessary. Therefore, attention should be given to COVID-19 vaccination during radiotherapy.

## Background

In December 2019, a case of unexplained pneumonitis in China was reported [1]. A disease caused by a coronavirus that has been named coronavirus disease 19 (COVID-19) has spread worldwide. More than 4 million deaths due to COVID-19 have been reported to the World Health Organization (WHO) in July 2021 [2]. To stop the COVID-19 pandemic, people need to keep social distancing, wear masks, and receive vaccination. Vaccination is essential for returning to a normal life. Vaccination has been progressing in developed countries and more than 3 billion doses of vaccines have been administrated worldwide [2]. The timing for receiving a vaccine and cancer treatment has sometimes become a problem. Among the various cancer treatment methods, it is not clear whether vaccines for patients receiving radiotherapy should be administered before, after, or during radiotherapy.

We experienced a patient who received a COVID-19 vaccine during radiotherapy and suffered from severe vertigo that resulted in the discontinuation of radiotherapy.

## Case presentation

**Fig. 1:**
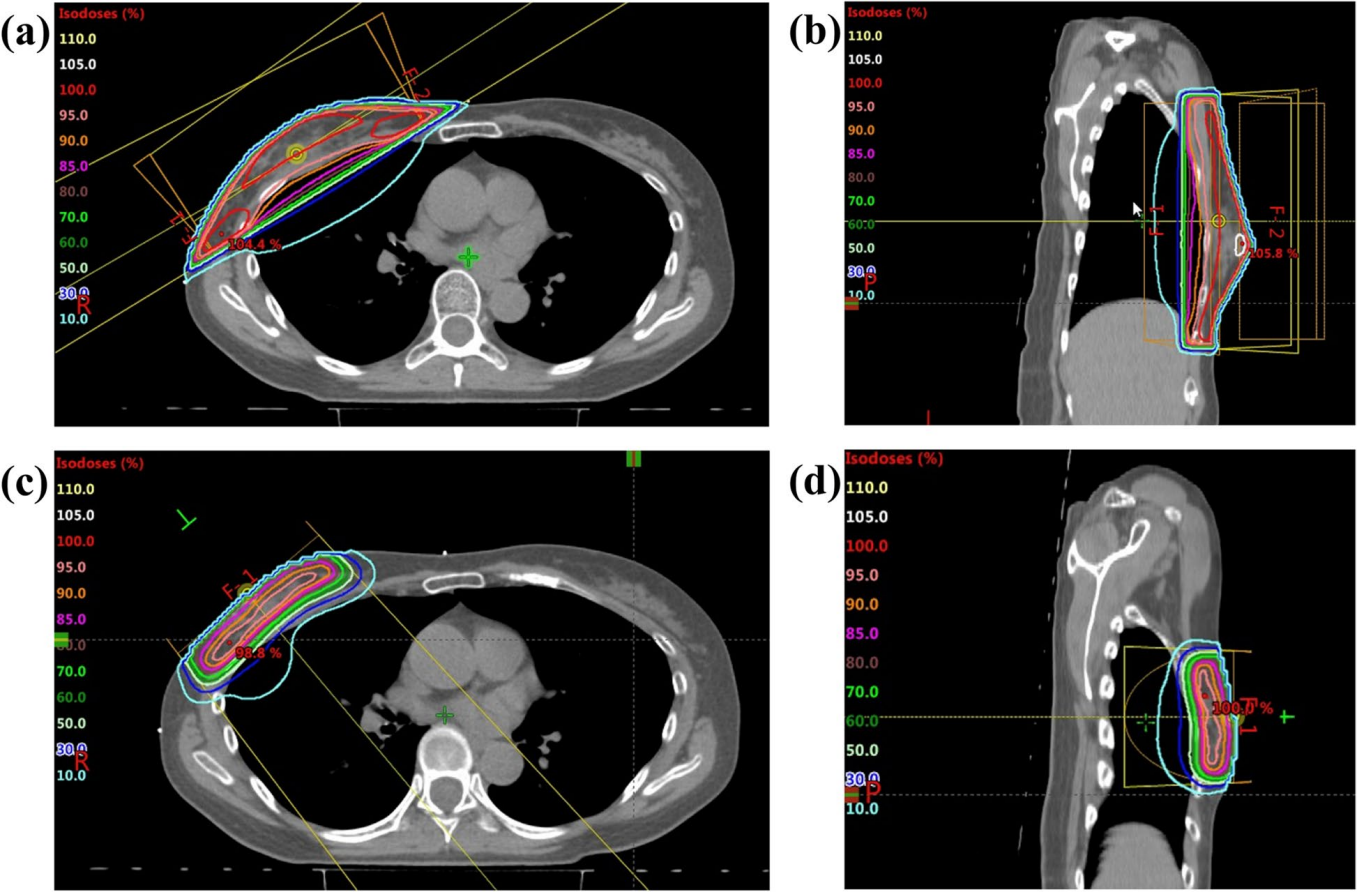
Radiotherapy fields. Axial (**a**) and sagittal (**b**) images of whole breast irradiation. Axial (**c**) and sagittal (**d**) images of tumor bed irradiation. Whole breast irradiation was performed at 2 Gy 25 times. Tumor bed irradiation was rescheduled from 5 times at 2 Gy to 4 times at 2.5 Gy due to vertigo

We report a 60-year-old Japanese woman with right breast cancer who received a COVID-19 vaccine during radiotherapy. She came to our department to receive postoperative radiotherapy. Her past medical history was shoulder pain, stomach ulcer, osteoporosis, and benign paroxysmal positional vertigo. She had an allergy to mackerel. She was diagnosed with noninvasive ductal carcinoma in the right breast. Partial mastectomy was performed 4 weeks before coming to our department. She had ductal carcinoma in situ, pathological TisN0M0, surgical margin close, estrogen-receptor-positive, progesterone-receptor-positive, human epidermal growth factor receptor type 2-negative and Ki-67 = 21.0%. She was scheduled to undergo irradiation at 2 Gy 25 times for the whole right breast followed by 2 Gy irradiation 5 times for the tumor bed (Fig. 1). Whole breast irradiation was performed with 6 MV X-ray. The body maximum dose was 106.7% (Fig. 1a, b). A COVID-19 vaccine (Tozinameran, Pfizer Inc, Germany) was administered in her left upper arm on day 3 and day 19 of the radiotherapy. She felt a slight pain in her left upper arm and a headache after the first vaccination. Computed tomography for radiation treatment planning was performed on day 12 because the body setup position was changed to reduce shoulder pain that had persisted from before the start of radiotherapy. She had a headache from day 14 and radiation dermatitis grade 1 (common terminology criteria for adverse events v5.0) from day 15. She received the second vaccine at day 19 of radiotherapy and suffered from physical weariness from the day after the second vaccination. Her systolic blood pressure was 190 and she suffered from grade 3 vertigo. She was hospitalized and diagnosed with Meniere’s disease at a local hospital. Radiotherapy was suspended for a total of 4 days. She came to our department using a wheelchair from day 22 and tumor bed irradiation was rescheduled for 4 times at 2.5 Gy in order to reduce the number of hospital visits. Tumor bed irradiation was performed using a 6 MeV electron beam without a bolus (Fig. 1c, d). Her vertigo continued during radiotherapy, but she managed to complete the radiotherapy. Her right breast skin became slightly red but she did not use any medication. Her treatment schedule and side effects are summarized in Table 1.

**Table 1:** Side effects and schedule of radiotherapy and vaccination

Day^a^	PS	Side effect	Event	Day^a^	PS	Side effect	Event
Day 1	0			Day 18	2	Headache	
Day 2	0			Day 19	2	Headache	Vaccination
Day 3	0		Vaccination	Day 20	2	Hypertension	Suspension of RT
Day 4	0	Arm pain		Day 21	2	Hypertension	Suspension of RT
Day 5	0			Day 22	2	Vertigo, hypertension	
Day 6	0			Day 23	2	Vertigo, hypertension	
Day 7	0			Day 24	3	Vertigo	Suspension of RT
Day 8	0			Day 25	3	Vertigo	Suspension of RT
Day 9	0			Day 26	3	Vertigo	
Day 10	1	Dullness		Day 27	3	Vertigo	
Day 11	1	Dullness		Day 28	3	Vertigo	
Day 12	0			Day 29	3	Vertigo	
Day 13	0			Day 30	2	Vertigo	
Day 14	1	Headache		Day 31	2	Vertigo	
Day 15	1	Headache, dermatitis^b^		Day 32	2	Vertigo	
Day 16	2	Headache		Day 33	2	Vertigo	
Day 17	2	Headache					

*RT *Radiotherapy, *PS *Performance status

^a^ The day was counted from the first day of radiotherapy and Saturday, Sunday and holidays were not counted

^b^ Dermatitis was continued, but she did not use any medication

She came to our department for a follow-up examination 4 weeks after the last day of radiotherapy. She was still suffering from vertigo. However, she could walk and had returned to work for a short time a few days before visiting our department. Radiation dermatitis was improving. The skin on her right breast was still slightly brown, but she had no pain or itching.

## Discussion

WHO reported in July 2021 that more than 3 billion vaccines had been administrated [2]. The safety of COVID-19 vaccination during radiotherapy is not clear. We experienced a patient who received a COVID-19 vaccine during radiotherapy. She suffered from severe vertigo and radiotherapy had to be suspended for 4 days. Fortunately, there is no clear evidence that the effectiveness of radiotherapy after partial mastectomy is reduced by suspension of radiotherapy for a few days. However, it had been reported that results in a worse treatment outcome in other cancer sites [3, 4]. Therefore, if patients receive a COVID-19 vaccine during radiotherapy, physicians need to consider the association between prolongation of radiotherapy duration and treatment outcomes.

Patients with cancer had more severe events of COVID-19 than did patients without cancer and that lung cancer was the most frequent cancer site [5]. Cancer patients with COVID-19 had a higher death rate, higher rate of admission to an intensive care unit, higher frequency of severe symptoms, and higher frequency of machine ventilation than those in non-cancer patients with COVID-19 [6]. Zhang et al. reported that 3 patients (10.7%) received chemotherapy, 1 patient (3.6%) received radiotherapy, 2 patients (7.1%) received molecular targeted therapy and 1 patient (3.6%) received immunotherapy within 14 days of the onset of COVID-19 among 28 COVID-19 patients with cancer [7]. Radiotherapy had less effect than drug therapy on COVID-19. Thus, patients with cancer have many disadvantages compared to patients without cancer.

Our patient received a Tozinameran vaccine (Pfizer Inc, Germany). Tozinameran is an mRNA vaccine and its effectiveness after 2 doses had been reported to be 95.3% [8]. Vaccine-related adverse events occurred in 21% of the participants who received Tozinameran and in 5% of the participants who received a placebo, and 2 participants who received Tozinameran and 4 participants who received a placebo died [9]. The participants in that study included participants with a shoulder injury, axillary lymphadenopathy, paroxysmal ventricular arrhythmia, and leg paresthesia, and participants under 55 years of age had more local and systemic adverse events than did participants aged 55 years or over. The people younger than 65 years of age had more local and systematic reactions than did people 65 years of age or older who received a Tozinameran or Moderna COVID-19 vaccine [10]. Our patient was 60 years old and it is therefore possible that she had greater adverse effects of the vaccine. She suffered from hypertension and severe vertigo. Hypertension and severe vertigo occurred after the second vaccination, and we speculate that these adverse events were associated with the vaccine. However, it is unclear whether radiotherapy made these adverse events more severe.

Partial mastectomy followed by radiotherapy is one of the common treatment strategies for breast cancer. Patients have to make many visits to a hospital for radiotherapy. Therefore, the COVID-19 Pandemic Breast Cancer Consortium has recommended a hypofractionated regimen in order to reduce the number of visits to a hospital [11]. Gasparri et al. reported that 48% of centers in 44 countries changed the radiotherapy procedure [12]. In their study, 22.6% of the centers postpone radiotherapy for patients with low risk, 23.3% of the centers treated patients with hypofractionated radiotherapy, and 2.1% of the centers changed the radiotherapy from a hospital-based procedure to an office-based procedure. Radiotherapy had to be suspended for patients with poor health conditions such as COVID-19 and adverse vaccine-related events during radiotherapy. Consideration should be given to hypofractionated radiotherapy in order to reduce such risks.

## Conclusions

Physicians need to consider that radiotherapy might need to be suspended for patients who receive COVID-19 vaccines during radiotherapy. Special attention is needed for patients under 65 years of age. If patients receive COVID-19 vaccines during radiotherapy, physicians need to consider the association between total radiotherapy duration and treatment outcomes.

## Data Availability

Not applicable

## References

[1] Huang C, Wang Y, Li X, et al. Clinical features of patients infected with 2019 novel coronavirus in Wuhan, China. Lancet. 2020;395(10223):497-506. 10.1016/S0140-6736(20)30183-5. 10.1016/S0140-6736(20)30183-5 PMC7159299 31986264

[2] The web site of the World Health Organization. https://www.who.int/emergencies/diseases/novel-coronavirus-2019?gclid=Cj0KCQjwweyFBhDvARIsAA67M71RnttAJ2p0UdpqdNtlE01PdNYcBels2nDH07mQouov2QVRlw1ouuAaApCCEALw_wcB

[3] D'Ambrosio DJ, Li T, Horwitz EM, et al. Does treatment duration affect outcome after radiotherapy for prostate cancer? Int J Radiat Oncol Bio Phys. 2008;72(5):1402–7. 18472368 10.1016/j.ijrobp.2008.03.011 PMC2763099

[4] Suwinski R, Sowa A, Rutkowski T, et al. Time factor in postoperative radiotherapy: a multivariate locoregional control analysis in 868 patients. Int J Radiat Oncol Biol Phys. 2003;56:399–412. 12738315 10.1016/s0360-3016(02)04469-3

[5] Liang W, Guan W, Chen R, et al. Cancer patients in SARS-CoV-2 infection: a nationwide analysis in China. Lancet Oncol. 2020;21(3):335–7. 32066541 10.1016/S1470-2045(20)30096-6 PMC7159000

[6] Dai M, Liu D, Liu M, et al. Patients with Cancer Appear More Vulnerable to SARS-CoV-2: A Multicenter Study during the COVID-19 Outbreak. Cancer Discov. 2020;10(6):783–91. 32345594 10.1158/2159-8290.CD-20-0422 PMC7309152

[7] Zhang L, Zhu F, Xie L, et al. Clinical characteristics of COVID-19-infected cancer patients: a retrospective case study in three hospitals within Wuhan. China. Ann Oncol. 2020;31(7):894–901. 32224151 10.1016/j.annonc.2020.03.296 PMC7270947

[8] Haas EJ, Angulo FJ, McLaughlin JM, et al. Impact and effectiveness of mRNA BNT162b2 vaccine against SARS-CoV-2 infections and COVID-19 cases, hospitalisations, and deaths following a nationwide vaccination campaign in Israel: an observational study using national surveillance data. Lancet. 2021;397(10287):1819–29. 33964222 10.1016/S0140-6736(21)00947-8 PMC8099315

[9] Fernando PP, Stephen JT, Nicholas K, et al. Safety and Efficacy of the BNT162b2 mRNA Covid-19 Vaccine. N Engl J Med. 2020;383(27):2603–15. 33301246 10.1056/NEJMoa2034577 PMC7745181

[10] Johanna CB, Julianne G, Tanya M. Reactogenicity Following Receipt of mRNA-Based COVID-19 Vaccines. JAMA. 2021;325(21):2201–2. 33818592 10.1001/jama.2021.5374

[11] Dietz JR, Moran MS, Idakoff SJ, et al. Recommendations for prioritization, treatment, and triage of breast cancer patients during the COVID-19 pandemic. the COVID-19 pandemic breast cancer consortium. Breast Cancer Res Treat. 2020;181(3):487–97. 32333293 10.1007/s10549-020-05644-z PMC7181102

[12] Gasparri ML, Gentilini OD, Lueftner D, et al. Changes in breast cancer management during the Corona Virus Disease 19 pandemic: An international survey of the European Breast Cancer Research Association of Surgical Trialists (EUBREAST). Breast. 2020;52:110–5. 32502796 10.1016/j.breast.2020.05.006 PMC7256546

